# Subchronic effects of different doses of Zinc oxide nanoparticle on reproductive organs of female rats: An experimental study

**DOI:** 10.18502/ijrm.v17i2.3988

**Published:** 2019-03-20

**Authors:** Seyed Mohammad Hosseini, Amir Hossein Moshrefi, Reza Amani, Seyed Vahid Razavimehr, Mohammad Hasan Aghajanikhah, Zahra Sokouti, Behnam Babaei Holari

**Affiliations:** ^1^Department of Pathology, Babol Branch, Islamic Azad University, Babol, Iran.; ^2^Young Researchers and Elite Club, Babol Branch, Islamic Azad University, Babol, Iran.; ^3^Department of Biology, Damghan Branch, Islamic Azad University, Damghan, Iran.

**Keywords:** *Zinc oxide*, *Nanoparticles*, *Ovary*, * Uterus*, * Toxicity.*

## Abstract

**Background:**

Zinc performs many biochemical and physiological functions; however, toxicological studies demonstrate that Nano-zinc oxide has harmful effects on human health and environmental species in high concentrations.

**Objective:**

The aim of this study was to investigate the toxicity of zinc oxide nanoparticles on reproductive tissues of female rat.

**Materials and Methods:**

Eighty female Wistar adult rats weighing 180–200 gr, divided into eight groups (n= 10 in each group) including control, sham (treated with saline), and six groups injected with different doses of zinc oxide nanoparticle with 10–30 nanometer size (4, 8, 25, 50, 100, and 200 mg/kg) twice a week for four weeks. At the end of the study, the rats were bled and slaughtered; the Ovary and Uterus were taken for histopathology studies and blood samples were transferred to the laboratory for biochemical analysis.

**Results:**

Microscopic diagnoses in ovary tissue were included; increase in the corpus luteum, follicular cysts, inflammatory cells infiltration and fibrosis. Histopathological changes in ovary in a dose-dependent manner. In uterus tissue the lesions consisted; epithelial destruction, hyperplasia of endometrial glands. The Estrogen and Progesterone level in the serum of rats increased in low doses and reduced in a dose-dependent manner at high doses.

**Conclusion:**

The results of the current study proved the toxicity of zinc oxide nanoparticles on the ovary and uterus organs at high concentrations, so further investigation is needed to reduce these effects.

## 1. Introduction

Heavy metals are relatively dense materials that exist in natural constituents of earth crust (1). In recent years, human activities such as industrial chemicals production and mining have increased the amounts of heavy metals into the environment and there has been increasing concern about their effects (1). In particular, zinc oxide (ZnO) nanoparticles (NPs) are commonly used to manufacturing products such as cosmetics, ceramics, plastics, glass, cement, rubber, grease, paints, pomade, glue and drug (2). The nanoparticle distribution in the environment depends on the properties of the metal including particle size, shape, surface characteristics, and various environmental factors (3). The evaluation of nanomaterials safety is of great importance for the industrial applications (4). It is also generally accepted that some of these metals are essentially required to retain various biochemical and physiological functions for living organisms in low amount, but toxic when they exceed their threshold concentrations (5). Many researchers investigated the potential side effect of Nano-sized particles and reported that prolonged exposure to environmental pollutants such as zinc can cause adverse health effects and harmful impacts on sexual function (1, 6). Exposure ZnO NPs during the organogenesis stage indicated poor embryonic development in mice. (7).

Also ZnO dissolution has been shown to be contributing to the acute or chronic toxicity of aquatic organisms (8). Heavy metals can be combined through food ingestion and inhalation and can influence the hypothalamic-pituitary-ovarian axis and play a role in the human fertility as a certain environmental factor (9). Mice treated with silica and titanium dioxide NPs had small uterus and fetuses (7). Jo and colleagues showed that exposure to Exposure to ZnO NPs prior to and during gestation could compromise the health of pregnant females and their fetuses. (8).

In addition heavy metals accumulation reduce progesterone and estradiol output. Pregnancies that occur despite a high heavy metal body load are at a greater risk of abortion, fetal deformity, premature birth and placental insufficiency (10).

The aim of this study was to investigate toxicity of ZnO NPs on reproductive tissues of female rat.

## 2. Materials and Methods

### Animal procedure

Eighty female Wistar rats of 8 weeks in age weighting 200±20 g were used in this study. They were obtained from North Research Center of Pasteur Institute of Iran (Amol, Iran) Research Center, Pasteur Institute of Iran, and were maintained in an environmentally controlled room (temperature, 20–22${}^{\circ }$C with 12 hr light/dark cycle) to gain desired body weight.

### Experimental design

In this Experimental study, the animals were divided into eight different experimental groups of 10 animals each: control: normal and apparently healthy rats that did not take any type of foodstuff. All other animals were received intraperitoneally (IP) twice a week for four weeks (1/2 ml for any animal) including: Group sham were given 10% normal saline body weight and the treatment group was given, respectively, 4, 8, 25, 50, 100, and 200 mg/kg body weight of ZnO nanoparticle Suspensions.

### Materials and preparation of nanoparticle suspension

**Figure 1:**
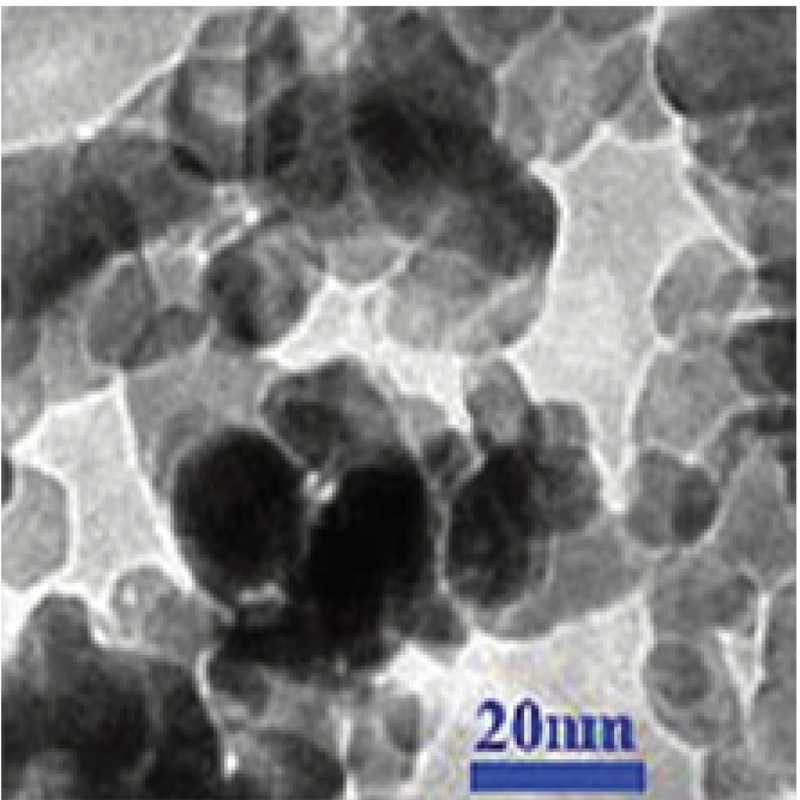
Scanning electron microscopy image of ZnO nanoparticles.

ZnO Nano powder was bought from the Iranian Nanomaterials Pioneers Company, NANOSANY (Mashhad, Iran). ZnO nanoparticle milky white color and 10–30 nm, other specifications are reported in Table I and transmission electron micrograph and crystal specifications of ZnO NPs are shown in Figure 1. An existing suspension was read by suspending ZnO NPs in *0.9*% *Saline*.

### Body weight and hormonal assay

At the end of the fourth week, the rats were anesthetized with intraperitoneal injection of xylazine and ketamine (20 and 60 mg/kg body weight, respectively, Alfasan, Woerden, Holland). Blood samples (3–5 cc) were taken from the heart of all the rats. Serum Estrogen and Progesterone hormones level was measured by using Elisa kit protocol (Sigma Aldrich, USA).

### Histopathological examination

From each animal, the sections of the ovaries and uterus were removed. The samples collected were fixed in 10% buffered formalin for 24 hr, and then routinely processed for histopathological examination and cut into 5-μm thick sections using a microtome (Leica, RM2235, Germany). Finally, the sections were stained with hematoxylin and eosin (H&E) (Sigma, England) and viewed under light microscope (CX31- OLYMPUS, Japan). A Tucsen TrueChrome Metrics camera and ISCapture software were used for histopathological evaluation.

### Evaluation of malondialdehyde (MDA)

Ovary homogenates were prepared by mixing 0.5 g of ovary tissue with 5 ml of phosphate buffer saline and homogenized by homogenizer (Polytron, Heidolph RZR 1, Germany). The suspension was centrifuged at 12,000×g for 15 min at 4${}^{\circ }$C (Biofuge Primo R, Heraeus, Germany). The supernatant was taken for measuring MDA. The samples were analyzed directly. This parameter has been measured using diagnostic Zell Bio kits and according to the manufactures recipes (Zell Bio, Germany).

### Ethical consideration

The experimental design adhered to the guidelines and was approved by the Animal Ethics Committee of Islamic Azad University, Babol branch, Babol, Iran.

### Statistical analysis

The data were analyzed using one-way analysis of variance, followed by post-hoc Duncan test by SPSS software version 22. A p< 0.05 was considered statistically significant.

## 3. Results

**Figure 2:**
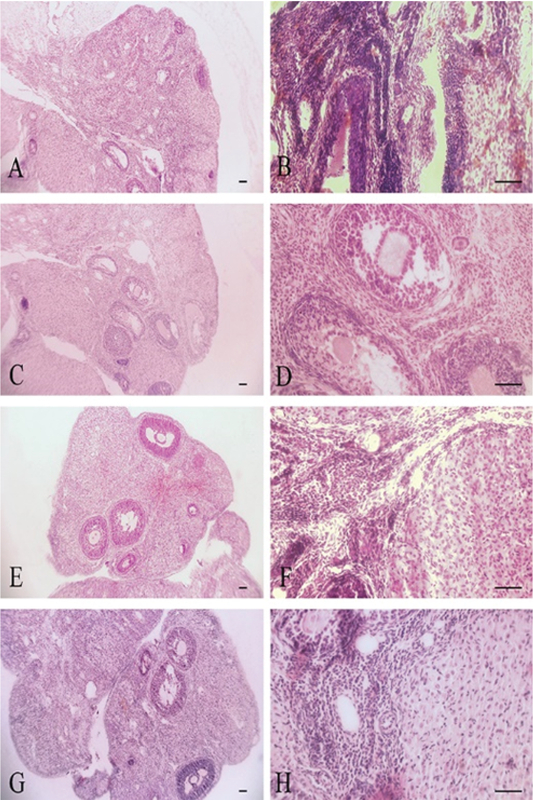
Ovary tissue. A, B: Control Group, Normal tissue. C, D: Sham Group, Normal tissue. E, F: 4 mg/kg, Normal tissue. G, H: 8 mg/kg, Normal tissue. H & E staining. X10, X40 Magnifications, scale bar= 50 μm.

**Figure 3:**
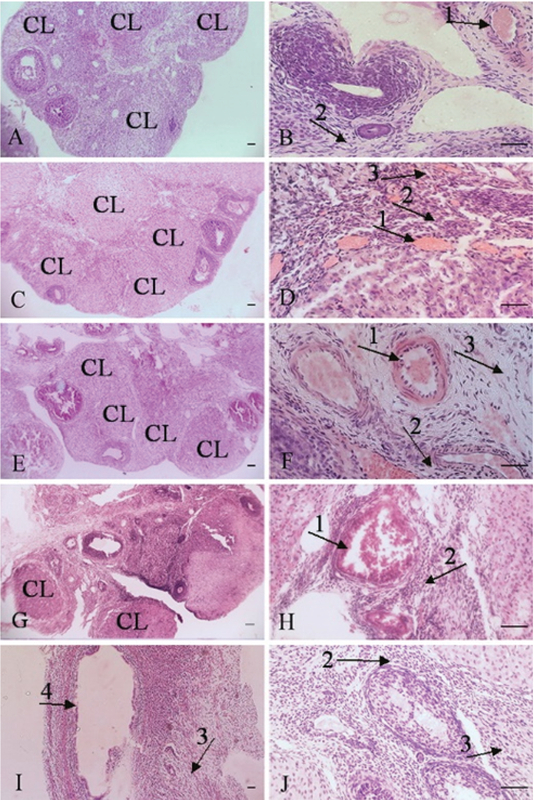
Ovary tissue. A, B: 25 mg/kg, Hyperemia (1), increased number of corpus luteum (CL), fibrosis (2). C, D: 50 mg/kg; E, F: 100 mg/kg, Hyperemia (1), increased number of corpus luteum (CL), infiltration of inflammatory cell (2), fibrosis (3). G, H, I, J: 200 mg/kg, Hyperemia (1), decreased number of corpus luteum (CL), infiltration of inflammatory cell (2), fibrosis (3), follicular cyst (4). H & E staining. X10, X40 Magnifications, scale bar= 50 μm.

**Figure 4:**
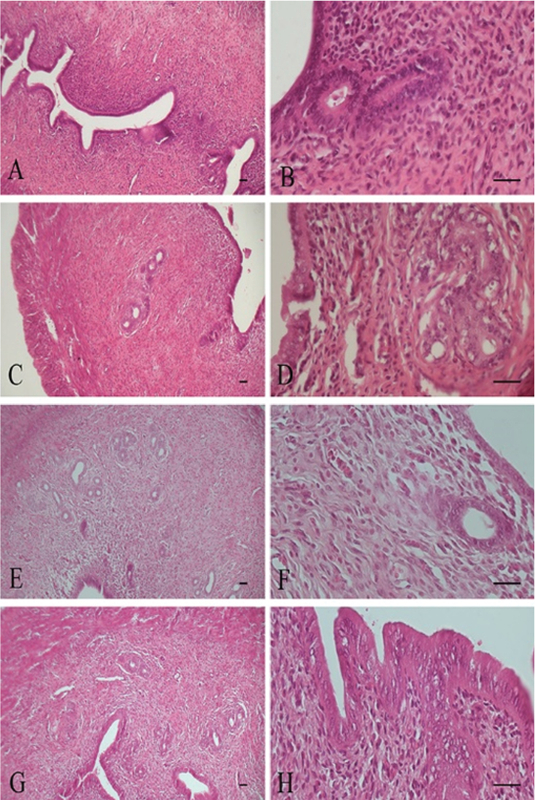
Uterus tissue. A, B: Control Group, Normal tissue. C, D: Sham Group, Normal tissue. E, F: 4 mg/kg, Normal tissue. G, H: 8 mg/kg, Normal tissue. H & E staining. X10, X40 Magnifications, scale bar= 50 μm.

**Figure 5:**
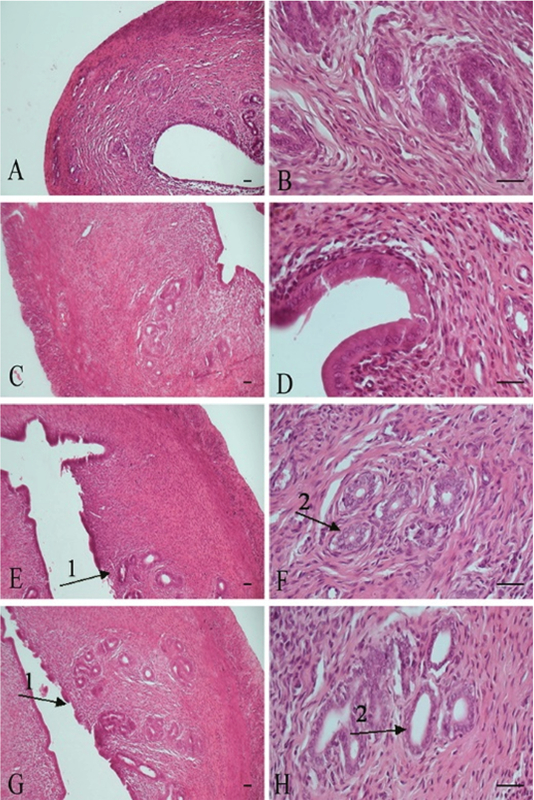
Uterus tissue. A, B: 25 mg/kg, Normal tissue. C, D: 50 mg/kg, Normal tissue E, F: 100 mg/kg: epithelial destruction (1), hyperplasia of endometrial glands (2). G, H: 200 mg/kg, epithelial destruction (1), hyperplasia of endometrial glands (2). H & E staining. X10, X40 Magnifications, scale bar= 50 μm.

Histopathological findings in ovary tissue included: Hyperemia, increase the corpus luteum, follicular cysts, inflammatory cells infiltration, and fibrosis (Table II, Figures 2 and 3). Histopathological findings in uterus tissue were epithelial destruction and hyperplasia of endometrial glands (Table II, Figures 4 and 5). Histopathological lesions in the ovary and uterus increased in a dose-dependent manner. The Estrogen and Progesterone level in the serum of rats after intraperitoneal injection of ZnO nanoparticle suspension are shown in Table III. The results showed that the exposure to ZnO nanoparticle dose-dependent increases the Estrogen level to 8 mg/kg and then reduces to 200 mg/kg (p< 0.0001). Progesterone level after increasing to 4 mg/kg reduces to 200 mg/kg, respectively (p< 0.004). There is a significant difference between groups shown in Table III. MDA levels increased dose-dependently in the treated group in ovary. There is a significant difference between treated groups at doses greater than 25 mg/kg (p< 0.0001) (Table III).

**Table 1:** Specifications of ZnO NPs.


**Characteristics**	**Structural color**	**Particle size (nm)**	**Specific Surface (m${}^{2}$/g)**	**True Density (g/sm${}^{3}$)**	**Purity (%)**
ZnO	Milky white	10–30	20–60	5/606	> 99%

**Table 2:** Pathological alteration of ovaries and uterus of rats exposed to ZnO NPs.


**Tissue**	**Ovary**				**Uterus**	
**Groups**	**Hyperemia**	**Inflammatory cell infiltration**	**Increase corpus Luteum**	**Fibrosis**	**Epithelial destruction**	**Gland hyperplasia**
Control	–	–	–	–	–	–
Sham	–	–	–	–	–	–
ZnO NPs dose						
4 mg/kg	–	–	–	–	–	–
8 mg/kg	–	–	–	–	–	–
25 mg/kg	+ +	+ +	–	–
50 mg/kg	+ +	+ +	–	–
100 mg/kg	++	++	+ ++	+ +
200 mg/kg	+ ++	–	++	+ ++

**Table 3:** Biochemical assay of serum and MDA level in the ovary exposed to ZnO NPs.


**Groups**	**Estrogen**	**Progesterone**	**MDA of Ovary nmol/mg**
Control	22.66 ± 1.84${}^{b,c}$	5.916 ± 0.56${}^{a,b}$	0.67 ± 0.03${}^{a}$
Sham	24.3 ± 1.59${}^{c,d}$	6.08 ± 0.48${}^{a,b}$	0.68 ± 0.03${}^{a}$
ZnO NPs dose			
4 mg/kg	28.46 ± 2.11${}^{d,e}$	8.44 ± 0.82${}^{c}$	0.678 ± 0.03${}^{a}$
8 mg/kg	33.86 ± 1.84${}^{e}$	8.19 ± 0.66${}^{c}$	0.68 ± 0.02${}^{a}$
25 mg/kg	31.99 ± 3.18${}^{e}$	7.44 ± 0.88${}^{b,c}$	1.06 ± 0.08${}^{b}$
50 mg/kg	31.19 ± 1.13${}^{e}$	6.02 ± 0.9${}^{a,b}$	1.6 ± 0.13${}^{c}$
100 mg/kg	18.42 ± 1.65${}^{a,b}$	5.68 ± 0.37${}^{a,b}$	2.73 ± 0.21${}^{d}$
200 mg/kg	16.42 ± 0.92${}^{a}$	5.23 ± 0.42${}^{a}$	3.59 ± 0.21${}^{e}$
Note: Data presented as mean ± SE; ${}^{a,b,c,d,e}$: Different letters a, b and c, etc., to show statistically significant differences between the groups.			

## 4. Discussion 

ZnO NPs is functional in many industries including dietary supplements in humans and livestock, food additives, pharmaceutics, and as a result of ultraviolet blocking features, they are also used in sunscreens, cosmetic, facial creams, ointments, lotions, and bottle coating. The wide use of NPs in different industries can lead to environmental contamination and indirect human exposure; and on the other hand due to its increasing usage in biological and medical applications such as cancer diagnosis and therapy, a person gets in direct exposure to these NPs (11, 12). Destructive effects on different organs and systems can be caused by short- and long-term exposure of heavy metals such as zinc. In the present study, female rats injected IP with ZnO NPs have shown light microscopic changes in the ovary and uterus due to ZnO NPs treatment. Histopathological results indicated dose-dependent increases in lesions with different doses of ZnO NPs injection; in the histopathological examination, the ovary in moderate and high doses treated with ZnO NPs showed hyperemia, increased the corpus luteum, inflammatory cells infiltration and fibrosis, follicular cysts and the uterus in high doses showed epithelial destruction and hyperplasia of endometrial glands. Ovary and uterus tissue of control group did not show any macroscopic and microscopic lesion. In contrast to the 200 (mg/kg) ZnO NPs-treated rats, the ZnO NPs content in the ovary and uterus of other groups' rats significant lesions were identified. A study by Mehta and co-worker reported arsenic-induced lesions in the ovary and uterus and decreased folliculogenesis that lead to reproductive failures (13). Lead (Pb) accumulated and damaged in the ovary and also has been found in sheep uterine tissues. High-dose Pb administration caused ovarian follicular cysts and led to poor corpus luteum. (14). Degeneration of uterine and decrease in primary follicles of ovaries were observed after sodium arsenite treatment. (15). Another study reported that the ovaries of Ni NPs treatment rats after gavage were associated with the infiltration of inflammatory cells, congestion, and ovarian lymphocytosis (16). Jo and colleagues reported that the uterus of adult female rats orally exposed to ZnO nanomaterials (500 mg/kg) showed pathological lesions such as localized necrosis, inflammation, thrombosis, and histiocyte infiltration (8). Oxidative stress is one of the major mechanisms behind heavy metal toxicity as measured by MDA content in vivo lipid peroxidation (17). Statistical analysis showed that MDA levels were significantly higher in treated groups compared to the control group. Our results point out that ZnO NPs generate free radicals that lead to cell damage. Following exposure to heavy metals such as cadmium and lead, metal were accumulated in ovary and can induce oxidative stress. (18). Suhartono and colleagues reported that exposure to Cd caused an increase in Advanced Oxidation protein products' level in rat kidney and increased MDA level in ovarian rats (19). In a study reported that following administration, Cd, as and Hg, serum MDA levels increased time and dose dependent. (20).

In the study of lipid peroxidation, MDA content in plasma, liver, and pancreas of exposed rats to Zinc was higher than that of ad libitum values (21). Chattopadhyay and co-worker showed that arsenic exposure led to a significant decrease of peroxidase activities in ovary and uterus. (15). A study showed that cadmium causes oxidative stress in kidney (19). The results of this study confirms that lipid peroxidation is one of the molecular mechanisms of cell injury in chronic ZnO NPs poisoning. Estrogen (E2) and Progesterone (P) are steroid hormones that have an important role in the reproductive tissues growth and differentiation, in the fertility, and maintain follicular development in general. These hormones are secreted of ovaries, so any destruction in ovary tissue can lead to their secretion disorder (22–24). In this study, Estrogen and Progesterone in serum were determined as well as ZnO concentrations in serum to investigate the effects of ZnO NPs. It has been reported that subchronic Aluminum exposure with medium and high doses causes a decrease in the levels of Estrogen and Progesterone in serum of the treated rats (25). In another study, Estrogen decreased after gavage administration of Ni NPs in adult rat (16). Low levels of ZnO NPs through stimulation of the glands cause increase in the Estrogen secretion. But high levels of ZnO NPs because of destructive effects on the ovary can cause decrease in the Estrogen secretion. It has been suggested that NPs have a greater risk of toxicity than larger particle (26), although the production and usage of these materials are increasing, the available information on the toxicological features about them are still incomplete.

## 5. Conclusion

ZnO NPs can enter animal and human tissues via air inhalation or food and manual handling; and they get absorbed in the tissues such as ovary. So because of the various usage and the possible harmful effects of ZnO NPs, investigating its toxicity is critical especially in mammalian cells. Due to changes in Estrogen and Progesterone and in the absence of lesions, it seems that low doses have positive effects.

##  Conflict of Interest

The authors declare that they have no conflict of interest.
